# High expression of AMAP1, an ARF6 effector, is associated with elevated levels of PD-L1 and fibrosis of pancreatic cancer

**DOI:** 10.1186/s12964-020-00608-8

**Published:** 2020-06-24

**Authors:** Akio Tsutaho, Ari Hashimoto, Shigeru Hashimoto, Soichiro Hata, Shion Kachi, Satoshi Hirano, Hisataka Sabe

**Affiliations:** 1 Department of Molecular Biology, Hokkaido University Faculty of Medicine, N15W7 Kitaku, Sapporo, 060-8638 Japan; 2 Department of Gastroenterological Surgery II, Hokkaido University Faculty of Medicine, N15W7 Kitaku, Sapporo, 060-8638 Japan; 3 Present Address: Laboratory of Immune Regulation, Immunology Frontier Research Center, Osaka University, 3-1 Yamadaoka, Suita, Osaka, 565-0871 Japan

**Keywords:** ARF6, AMAP1, Fibrosis, FAK, Pancreatic cancer, PD-L1

## Abstract

**Background:**

Not merely the onset of immune evasion, but other factors, such as acidosis and fibrosis, are also major barriers in cancer therapeutics. Dense fibrosis is a hallmark of pancreatic ductal carcinoma (PDAC), in which hyperactivation of focal adhesion kinase (FAK) in tumor cells was shown to be crucial. Double mutations of *KRAS/ TP53* are characteristic to PDAC. We previously showed that high protein expression of ARF6 and its downstream effector AMAP1, as well as processes involved in the ARF6 activation by cell surface tyrosine kinase receptors, are major targets of the *KRAS*/*TP53* mutations to promote PDAC invasion, metastasis, and immune evasion. This notion was recaptured by KPC mouse model of human PDAC (*LSL-Kras(G12D/+); LSL-Trp53(R172H/+)); Pdx-1-Cre*). Mechanistically, the ARF6-AMAP1 pathway is primarily involved in cellular dynamics of PD-L1, β1-integrins, and E-cadherin; and hence modulates cell-adhesion properties when ARF6 is activated. Here, with an aim to understand whether the ARF6-AMAP1 pathway is critically involved in the elevated levels of PD-L1 and fibrosis of PDAC, we analyzed relationship between AMAP1 and these malignant phenotypes. Moreover, because the ARF6 pathway may closely be related to focal adhesion dynamics and hence to FAK, we also investigated whether AMAP1 employs FAK in fibrosis.

**Methods:**

Clinical specimens, as well as KPC cells/tumors and their *shAMAP1* or *shFAK* derivatives were analyzed.

**Results:**

Elevated levels of PD-L1 and fibrosis correlated with poor outcome of our patient cohort, to be consistent with previous reports; in which high AMAP1 expression statistically correlated with the elevated PD-L1 and fibrosis. To be consistent, silencing of *AMAP1* (*shAMAP1*) in KPC cells resulted in reduced PD-L1 expression and fibrosis in their tumors. On the other hand, *shAMAP1* only slightly affected FAK activation in KPC cells, and phosphorylated FAK did not correlate with enhanced fibrosis or with poor outcome of our patients.

**Conclusions:**

Together with our previous data, our results collectively indicated that the ARF6-AMAP1 pathway, empowered by the *KRAS/TP53* mutations, is closely associated with elevated PD-L1 expression and fibrosis of human PDACs, to be recaptured in the KPC mouse model. The ARF6 pathway may promote fibrosis independent of FAK.

Video abstract

## Background

The onset of immune checkpoints, including the expression and signaling of programmed cell death protein-1/programmed death ligand-1 (PD-1/PD-L1), is not the complete mechanism driving tumor immune evasion, and other factors, including metabolic intercommunication or competition between tumor cells and stroma [1], acidosis of the tumor microenvironment (TME) [2], hydrolysis of microenvironmental ATP [3], and tumor fibrosis [4], are also amongst the major obstacles.

Dense fibrosis is a hallmark of pancreatic ductal carcinoma (PDAC). Fibrosis may affect both tumor cells and cells in the TME, and often demonstrate duality in cancer biology and therapeutics [5]. For example, abatement of fibrosis by depleting cancer-associated fibroblasts in PDAC mouse models results in TME immunosuppression with decreased animal survival [6]. However, although fibrosis is not a simple physical barrier, its abatement might be a prerequisite to improve checkpoint immunotherapy by enabling the efficient access of immune cells to tumor cells [6, 7].

In PDAC, tumor cells on their own greatly contribute to the enhanced fibrosis, in which the high expression and hyperactivation of focal adhesion kinase (FAK) in tumor cells was shown to be crucial [7] (In the present paper, FAK refers to FAK1, not PYK2/FAK2). FAK is a non-receptor protein tyrosine kinase and is primarily activated upon the binding of integrins to the extracellular matrix (ECM). Both FAK expression and activation are upregulated in the tissue of more than 80% of PDAC patients compared with that in normal pancreatic tissue, and tightly correlated with enhanced fibrosis [7], in which FAK activation was assessed by the immunostaining of Tyr-397 phosphorylation (p-FAK). Interestingly, these FAK-associated events also correlate with the immunosuppressive TME, that is, lower numbers of tumor-infiltrating CD8-positive T-cells and higher amounts of neutrophil elastase-positive and CD15-positive granulocytes [7]. Moreover, FAK-associated events were shown to correlate with immunosuppressive cytokine production, by yet unknown mechanisms [7]. On the other hand, it is well documented that not merely the total amounts of integrins involved in cell-substratum adhesion, but the physical tension of the ECM toward integrins is also crucial in activating FAK [8]. Likewise, the abundance of collagen in PDAC has been controversial in its association with patient prognosis [9–12], and the deposition of specific types of collagen, such as collagen I, was reported to more closely correlate with the poor prognosis of patients [7, 13].

ARF6 is a small GTPase that is primarily involved in the outward flow of plasma membrane components. A series of our studies demonstrated that ARF6 and one of its downstream effectors, namely, AMAP1 (also called DDEF1 and ASAP1), constitute the core signaling machinery that drives cancer malignancy and therapeutic resistance when ARF6 is activated. ARF6 and AMAP1 proteins are often abnormally overexpressed in different types of cancers, including breast cancer, renal cancer, lung adenocarcinoma, head and neck cancer and PDAC, to be statistically correlated with the rapid recurrence and poor overall survival of patients [14–23]. In PDAC, *KRAS* and *TP53* mutations are known to be major driver oncogenes, in which mutations in *KRAS* occur in 90–95% and mutations in *TP53* in up to 75% [24–27]. We have identified that the *KRAS* oncogene causes the overexpression of ARF6 and AMAP1 proteins via upregulating eukaryotic initiation factor 4A/4E (eIF4A/4E) activities to promote the translation of their mRNAs, whereas the *TP53* oncogene facilitates processes involved in ARF6 activation [23], in which *TP53* acts to upregulate the expression of mevalonate pathway enzymes and platelet-derived growth factor receptor (PDGFR) β [19, 28]. Mechanistically, AMAP1 has several different protein-protein interaction modules [16], and is thereby linked to various different cellular events, including the remodeling of cortical actin structure, turnover of focal adhesions, recycling of β1 integrins and PD-L1, and endocytosis of E-cadherin [16, 23, 29, 30]. Thus, the ARF6-AMAP1 pathway appears to regulate cell adhesion avidities towards ECMs or towards other cells. The ARF6-AMAP1 pathway is moreover involved in intracellular transport of mitochondria, in order to avoid unnecessary production of superoxides [31]. Owing to this activity, cancer cells overexpressing the ARF6 pathway demonstrate robust resistance against ionizing radiation [31]. We have furthermore found that this pathway also promotes immune evasion of PDAC in vivo (23; see below).

KPC mouse cells are the representative model for human PDAC and have *KRAS* and *TP53* double mutations (*LSL-Kras(G12D/+); LSL-Trp53(R172H/)); Pdx-1-Cre*) [32]. We have shown that KPC cells express ARF6 and AMAP1 at high levels, and that the ARF6 pathway is involved in recycling of PD-L1 in vitro, as well as an immune evasion phenotype of KPC cells in vivo [23]. Herein, by using KPC cells/tumors and human clinical specimens, we aimed to understand whether the ARF6-AMAP1 pathway is involved in the elevated levels of PD-L1 and fibrosis of PDAC.

## Materials and methods

### Antibodies and chemicals

Affinity-purified rabbit polyclonal antibody against human AMAP1 was as described previously [16]. This antibody cross-reacts with mouse AMAP1. Other antibodies were purchased from commercial sources, as follows: rabbit monoclonal antibodies against PD-L1 (Cell Signaling) and phospho-FAK (Thermo Fisher Scientific), rabbit polyclonal antibody against PD-L1 (Novus), rabbit polyclonal antibody against collagen I (Proteintech), rabbit polyclonal antibody against FAK [33], mouse monoclonal antibody against β-actin (Sigma-Aldrich). Donkey antibodies against rabbit or mouse IgGs, each conjugated with horseradish peroxidase, were from Jackson ImmunoResearch Laboratories. Immunoblotting analysis was performed using the ECL kit (GE Healthcare), as described previously [15].

### Cells

KPC cells were a gift from Y. Kodama and Y. Nishikawa (Kyoto University Graduate School of Medicine; see 23), and were cultured in Dulbecco’s modified Eagle’s medium (DMEM; Sigma-Aldrich) containing 10% fetal calf serum (HyClone, Thermo Fisher Scientific) at 37 °C with 5% CO_2_. KPC cells expressing shRNAs against *AMAP1* (#1 and #2) and a control scramble shRNA (Irr) were described previously [23]. shRNA lentiviral vectors against *FAK* were purchased from Sigma-Aldrich. KPC cells were transduced with these *shFAKs* (#1 and #2), as described previously [19]. Cell viabilities were measured using Cell Counting Kit-8 (Dojindo), according to the manufacturer’s instructions.

### Patients and tissue samples

All specimens were selected from patients who underwent pancreatectomy at Hokkaido University Hospital between January 2000 and December 2011, and were analyzed retrospectively. None of the patients received chemotherapy or radiation therapy before surgery. Clinicopathological parameters of the patients at the time of pancreatectomy are summarized in Table 1. This study was approved by the Institutional Review Board of Hokkaido University Hospital (study approval no. 017–0059). Comprehensive agreement regarding specimen storage was obtained in writing from all patients at the time. PDAC is one of the most aggressive types of solid malignancies. In particular, the 5-year survival rate remains low at approximately 5 to 7%. Therefore, at the beginning of our study, these patients had passed away or were unable to give informed consent. However, the Institutional Review Board of Hokkaido University Hospital recognized the importance of analyzing these clinical specimens in our study and approved this study without requiring written consent from individual patients.

**Table 1:** General characteristics of the patients whose samples were analyzed by tissue micro array analysis (*n* = 164)

	n (%)
Sex	
Male	96 (58.5%)
Female	68 (41.5%)
Age, yr	65 (43–89)^b^
Tumor location	
Head^a^	94 (57.3%)
Body + tail	70 (42.7%)
Tumor size, cm	3.0 (1.0–8.0)^b^
Surgical margin	
Positive	23 (14.0%)
Negative	141 (86.0%)
pT	
1 + 2	7 (4.3%)
3 + 4	157 (95.7%)
Regional lymph node metastasis	
Positive	125 (76.2%)
Negative	39 (23.8%)
Distant metastasis	
Positive	12 (7.3%)
Negative	152 (92.7%)
Pathological stage	
IA-IIA	38 (23.2%)
IIB-IV	126 (76.8%)
Lymphatic invasion	
Positive	107 (65.2%)
Negative	57 (34.8%)
Vascular invasion	
Positive	135 (82.3%)
Negative	29 (17.7%)
Perineural invasion	
Positive	144 (88.4%)
Negative	20 (11.6%)
Adjuvant chemotherapy ^c^	
Positive	83 (50.6%)
Negative	80 (48.8%)
Recurrent disease^d^	
Positive	131 (79.9%)
Negative	31 (18.8%)

^a^One pancreas head tumor metastasized to other parts of the body

^b^Values represent the median (range)

^c^Information regarding adjuvant chemotherapy could not be obtained for one patients

^d^Information regarding recurrence could not be obtained for two patients

### Immunohistochemical staining (IHC) and sirius red staining

IHC analyses of tissue microarrays were performed, as described previously [16]. Briefly, samples were deparaffinized in xylene and dehydrated in a graded series of ethanol. After rinsing in TBS buffer, the samples were processed for antigen retrieval in a 1 mM EDTA retrieval solution (pH 9.0) (Nichirei Co.) at 95 °C for 40 min. Endogenous peroxidase was then blocked by incubation in 0.3% H_2_O_2_-methanol at room temperature for 10 min. After rinsing with TBS, samples were incubated with primary antibodies against human PD-L1 (1:200) and p-FAK (1:100) for 30 min, and then with EnVision (Dako Corp) for 30 min. After rinsing in TBS, the coloring reaction was performed with DAB (Dojindo) for 5 min. Each sample was counterstained with hematoxylin. These processes were all performed at room temperature. For sirius red staining, tissue slides were dewaxed, dehydrated, and stained with Picro-sirius red for 1 h (Sigma-Aldrich, S365548). After staining, slides were washed in two dips of acidified water and dehydrated with 100% ethanol. Finally, slides were cleared in xylene and mounted. Quantification of collagen staining was performed using Image J software [7].

### Evaluation of IHC staining

At least two microarray tissue cores of each clinical sample were subjected to IHC, and the results were evaluated by two clinical doctors independently using a light microscope at a magnification of × 200. Staining intensities were graded on a scale from 0 to 3 (0, no staining; 1, weak staining; 2, medium staining; and 3, strong staining); and percentages of the stained areas were calculated as proportions (0 if 0%, 0.1–0.9 if 10–90%, respectively, and 1 if 100%). Proportional scores were then multiplied by the staining grades to obtain H scores. The final H score of each sample was indicated as an average H score of each corresponding core. For PD-L1 staining, strong PD-L1 immunostaining in more than 10% of neoplastic cells was classified as PD-L1 positive, and in less than 10% was classified as PD-L1 negative. The overall survival of patients was defined as the interval between surgery and death, or between surgery and the last observation point. For surviving patients, data were censored at the last follow up. Disease-free survival was defined as the interval between the date of surgery and the date of diagnosis of any type of relapse.

### Flow cytometric analysis

KPC cells pretreated with shRNAs were incubated with IFNγ (50 ng mL^− 1^) for 48 h. Cells were then detached from culture dishes using 4 mM EDTA in PBS, collected by centrifugation, and incubated with either PE-conjugated anti-mouse PD-L1 antibody (Clone: MIH5, eBioscience) or an isotype-matched antibody in PBS with 2% FCS for 40 min on ice. After washing with PBS, cells were then incubated with 7AAD (BD Pharmingen) in PBS for 10 min on ice, and subjected to flow cytometric analysis (BD FACSVerse™).

### Tumor cell inoculation

All animal experiments were performed according to a protocol approved by the Animal Care Committee of Hokkaido University. KPC cells (2 × 10^5^) in 100 μL of 50% Growth Factor Reduced BD Matrigel Matrix (BD Bioscience) were injected into flank of C57/BL6 mice (8 to 10-week old females, CLEA Japan) and tumor tissues were collected on day 14.

### Statistical analysis

Comparisons between two groups were performed using the *t*-test. Differences in survival between groups were analyzed by the log-rank test. All statistical analyses were conducted using the JMP for Windows version 12.0 software package (SAS Institute Inc., Cary, NC, USA).

## Results

**Fig. 1:**
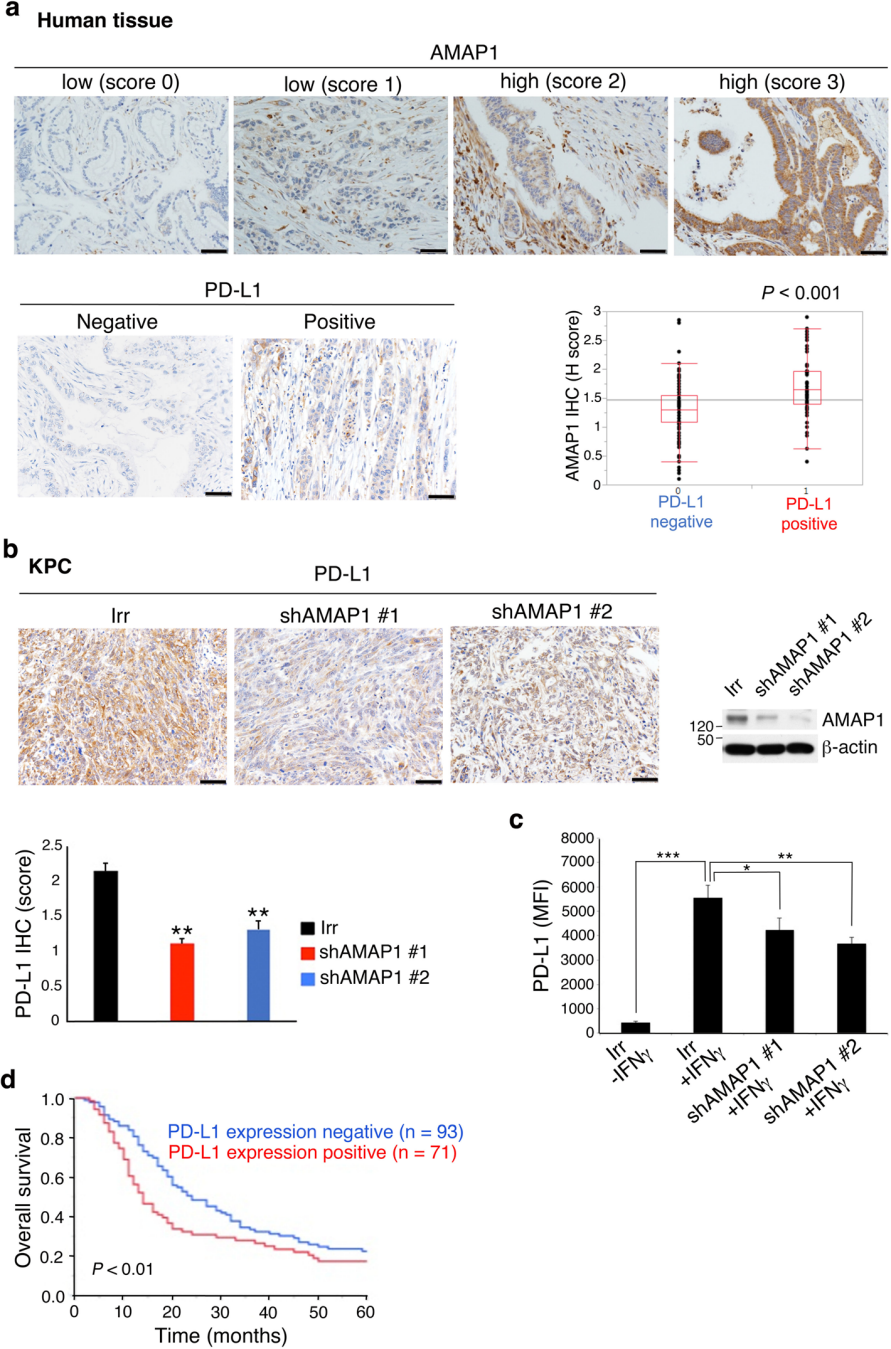
High AMAP1 expression levels statistically correlate with PD-L1 expression in human PDACs and KPC tumors. **a** Representative IHC images of AMAP1 with IHC scores 0 to 3 and positive and negative staining of PD-L1 in clinical specimens, and their comparison with AMAP1 IHC scores. **b** IHC images and quantification of the PD-L1 staining of tumors formed by control (Irr) or *AMAP1*-silenced (*shAMAP1* #1 and #2) KPC cells in C57BL/6 mice. Error bars represent the mean ± s.e.m. **P < 0.01. **c** PD-L1 cell surface expression in IFNγ-treated or non-treated KPC cells, pretreated with shRNAs. MFI, median fluorescence intensity. Error bars represent the mean ± s.e.m. *P < 0.05, **P < 0.01, ***P < 0.001. **d** Kaplan-Meier plots of the overall survival of patients with regard to PD-L1 positivity. *P*-values were obtained by *t*-tests (**a**, **b** and **c**) and by the log-rank test (**d**). Bars = 100 μm (**a** and **b**)

We previously showed that the ARF6-AMAP1 pathway promotes the recycling of PD-L1, and that blockade of the ARF6 pathway significantly reduces the cell-surface expression of PD-L1 in vitro [23]. In this study, we first sought to confirm the association between the ARF6 pathway and PD-L1 in humans using resected specimens and their immunohistochemical staining. However, ARF6 is a small GTP-binding protein and its immunohistochemical staining was difficult. On the other hand, we have generated antibodies that clearly label AMAP1 of clinical specimens [16, 20, 23]. By immunohistochemical staining, we found that high levels of AMAP1 statistically correlate with high levels of PD-L1 (Fig. 1a and Table 1; see ref. [23] for AMAP1 staining and expression). Consistently, tumors formed by KPC cells in which *AMAP1* was silenced by shRNA plasmids (#1 and #2) showed significantly reduced PD-L1 staining compared with those of parental KPC tumors (Fig. 1b). Cell surface levels of PD-L1, as measured using a fluorescence-activated cell sorter, were also decreased upon *AMAP1* silencing (Fig. 1c). Therefore, our results revealed a close association between the high AMAP1 expression and the elevated PD-L1 levels in human PDACs, to be recaptured in the KPC mouse model. Furthermore, consistent with a previous report [34], higher expression levels of PD-L1 statistically correlated with poor overall survival of our patient cohort (Fig. 1d; see *Materials and methods* for PD-L1 scoring).

**Fig. 2:**
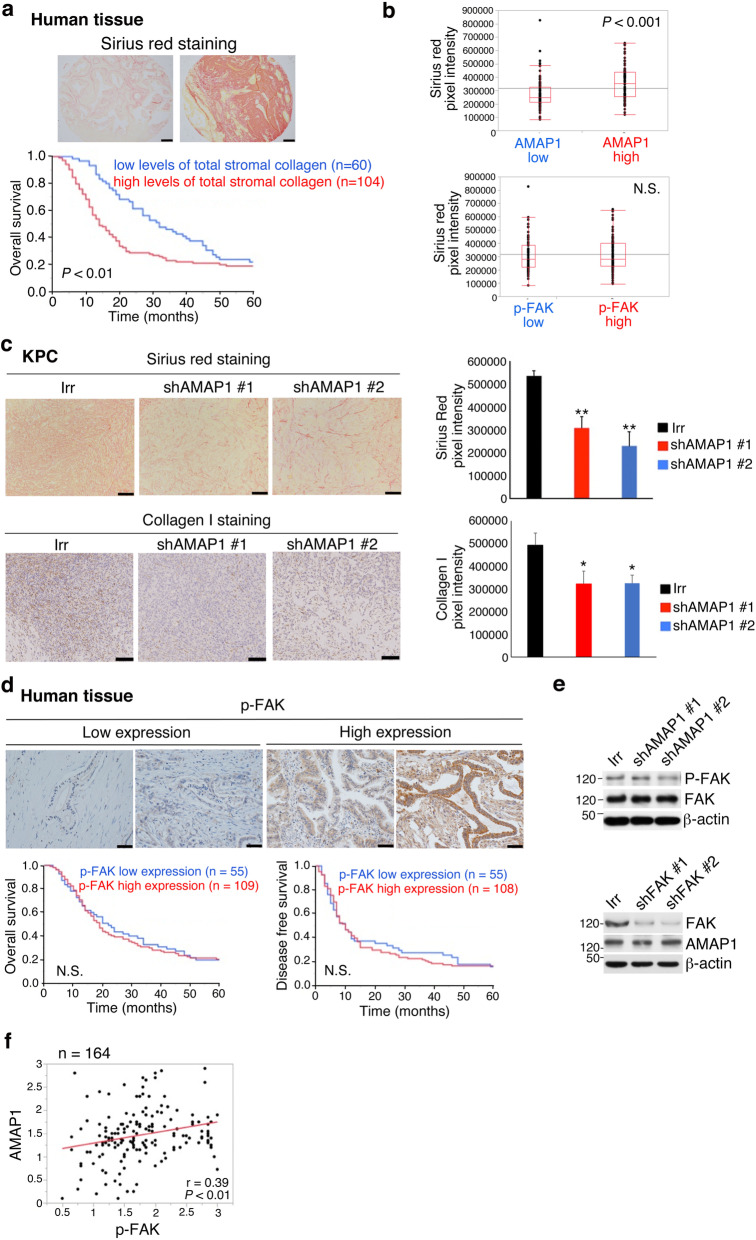
High AMAP1 expression levels statistically correlate with fibrosis in human PDACs and KPC tumors. **a** Representative images of sirius red staining of clinical specimens, and Kaplan-Meier plots of overall survival of patients with regard to the staining. Bars = 200 μm. **b** Comparison of sirius red staining intensities in human samples with high or low AMAP1 expression or phosphorylated FAK (p-FAK) levels. **c** Representative images and quantification of sirius red and collagen I staining of tumors formed by control (Irr) or *AMAP1*-silenced (*shAMAP1* #1 and #2) KPC cells in C57BL/6 mice. Error bars represent the mean ± s.e.m. Bars = 100 μm. **d** Representative IHC images of p-FAK and Kaplan-Meier plots with regard to the different levels of p-FAK in human PDACs. Bars = 50 μm. **e** Immunoblot analysis of FAK, p-FAK, and AMAP1 levels in control (Irr) and *shFAK* or *shAMAP1* KPC cells, after culturing for 40 h in vitro. β-actin was used as a control. **f** A positive correlation between AMAP1 and p-FAK expression levels in human PDACs. *P < 0.05, **P < 0.01. *P*- values were obtained by the log-rank test (**a** and **d**) and by *t*-tests (**b**, **c**, and **f**)

Higher sirius red staining of PDACs (i.e., dense tumor fibrosis) also statistically correlated with the poorer patient survival of our cohort (Fig. 2a), to be consistent with a previous report [7]. Moreover, the elevated levels of sirius red staining in our clinical samples statistically correlated with the high AMAP1 levels (Fig. 2b). We confirmed this relationship with the KPC mouse model, in which silencing of *AMAP1* in KPC cells (#1 and #2) results in significantly reduced staining of sirius red and collagen I in their tumors (Fig. 2c).

The blockade of FAK in KPC cells, either by *siFAK* or by an FAK inhibitor, has been shown to reduce tumor fibrosis [7], whereas the ARF6-AMAP1 pathway may promote focal adhesion dynamics depending on cellular conditions, as mentioned earlier. We then analyzed the possible association between FAK and AMAP1-mediated fibrosis. Unlike in the case of AMAP1, p-FAK levels did not correlate with sirius red staining density in PDACs of our patient cohort (Fig. 2b). P-FAK levels also did not correlate with the poor outcome of our patients (Fig. 2d). We also investigated cultured KPC cells, and found that the silencing of *AMAP1* appeared to only slightly, if any, reduce p-FAK levels, and that the silencing of FAK did not affect AMAP1 expression (Fig. 2e). Likewise, p-FAK levels demonstrated only a weak correlation with the AMAP1 levels of clinical samples (Fig. 2f). Therefore, FAK does not appear to be at the core of the AMAP1-mediated tumor fibrosis and malignancy.

## Discussion

In this paper, we showed that high AMAP1 protein expression statistically correlate with high expression of PD-L1 in clinical specimens of PDAC. We also showed that the high AMAP1 protein expression statistically correlate with enhanced fibrosis in clinical specimens of PDAC. Both of these correlations were clearly recaptured in KPC tumors, the representative mouse model of human PDAC.

Although we showed that the AMAP1 overexpression correlates with the PD-L1 levels, we previously showed that the ARF6-AMAP1 pathway primarily regulates the intracellular recycling of PD-L1, but not its gene expression or protein expression [23]. It has been shown that certain types of cell-surface proteins are designated to be degraded, such as by lysosomes, unless they are actively recycling [35]. Thus, it can be assumed that long-term blockade of PD-L1 dynamics via inhibition of the ARF6-AMAP1 pathway by *shAMAP1* might lead to its protein degradation, although we are yet to analyze this in detail. Moreover, PD-L1 may also be expressed in cells other than tumor cells such as myeloid-derived suppressor cells of TME, including in the case of pancreatic cancer [36]. Our IH analysis of this study did not precisely distinguish whether PD-L1 is expressed at tumor cells or other cells. Thus, whether activation of the ARF6-AMAP1 pathway of tumor cells affects PD-L1 expression of non-tumor cells also awaits to be clarified.

We also previously showed that *shAMAP1* significantly reduces the immune evasive phenotype of KPC cells in vivo [23]. Likewise, FAK was shown to be crucially involved in immune evasion, as well as the fibrosis of KPC tumors [7]. Furthermore, the ARF6-AMAP1 pathway can be involved in focal adhesion dynamics [29, 31]. Thus, we were interested in analyzing whether AMAP1 utilizes FAK. However, our results suggested that AMAP1 is dispensable for FAK activation in KPC cells, and that AMAP1 levels are not tightly associated with p-FAK levels in clinical samples. Moreover, whereas high AMAP1 expression levels correlate with poor patient outcomes, high p-FAK expression levels did not show a significant correlation with poor patient outcomes in our cohort. On the other hand, besides fibrosis, p-FAK was also shown to be closely associated with the immunosuppressive properties of the TME, by yet unknown mechanisms [7]. Likewise, we still do not know the entire mechanism by which the ARF6-AMAP1 pathway promotes tumor immune evasion, as well as fibrosis.

In other words, it awaits to be clarified as to whether the enhanced expression and dynamics of PD-L1, as well as enhanced fibrosis are the complete mechanism by which the ARF6-AMAP1 pathway promotes tumor immune evasion, or whether some other unidentified mechanisms exist that are also driven by this pathway.

## Conclusion

Elevated levels of PD-L1 and fibrosis of PDACs are remarkable risk factors for the patient poor outcome. In this study, we demonstrated that the ARF6-AMAP1 pathway is critically involved in the elevated levels of PD-L1 and fibrosis of human PDACs, to be recaptured experimentally with the KPC mouse model.

## Data Availability

All data generated during this study are included in this article.

## Abbreviations

ECM: Extracellular matrix
eIF4A/4E: Eukaryotic initiation factor 4A/4E
FAK: Focal adhesion kinase
IHC: Immunohistochemical staining
PDAC: Pancreatic ductal carcinoma
PDGFR: Platelet-derived growth factor receptor
PD-1/PD-L1: Programmed cell death protein-1/programmed death ligand-1
s.e.m.: Standard error of the mean
TME: Tumor microenvironment

## References

[1] Chang CH (2015). Metabolic competition in the tumor microenvironment is a driver of cancer progression. Cell.

[2] Pillai SR (2015). Causes, consequences, and therapy of tumors acidosis. Cancer Metastasis Rev.

[3] Allard B (2015). The ectonucleotidases CD39 and CD73: novel checkpoint inhibitor targets. Immunol Rev.

[4] Jiang H, Hegde S, DeNardo DG (2017). Tumor-associated fibrosis as a regulator of tumor immunity and response to immunotherapy. Cancer Immunol Immunother.

[5] Chandler C (2019). The double edge sword of fibrosis in cancer. Transl Res.

[6] Özdemir BC (2014). Depletion of carcinoma-associated fibroblasts and fibrosis induces immunosuppression and accelerates pancreas cancer with reduced survival. Cancer Cell.

[7] Jiang H (2016). Targeting focal adhesion kinase renders pancreatic cancers responsive to immunotherapy. Nat Med.

[8] Tilghman RW, Parsons JT (2008). Focal adhesion kinase as a regulator of cell tension in the progression of cancer. Semin Cancer Biol.

[9] Dangi-Garimella S (2011). Three-dimensional collagen I promotes gemcitabine resistance in pancreatic cancer through MT1-MMP-mediated expression of HMGA2. Cancer Res.

[10] Erkan M (2008). The activated stroma index is a novel and independent prognostic marker in pancreatic ductal adenocarcinoma. Clin Gastroenterol Hepatol.

[11] Sinn M (2014). α-Smooth muscle actin expression and desmoplastic stromal reaction in pancreatic cancer: results from the CONKO-001 study. Br J Cancer.

[12] Bever KM (2014). The prognostic value of stroma in pancreatic cancer in patients receiving adjuvant therapy. HPB (Oxford).

[13] Laklai H (2016). Genotypes tunes pancreatic ductal adenocarcinoma tissue tension to induce matricellular fibrosis and tumor progression. Nat Med.

[14] Sabe H (2003). Requirement for Arf6 in cell adhesion, migration, and cancer cell invasion. J Biochem.

[15] Hashimoto S (2004). Requirement for Arf6 in breast cancer invasive activities. Proc Natl Acad Sci U S A.

[16] Onodera Y (2005). Expression of AMAP1, an ArfGAP, provides novel targets to inhibit breast cancer invasive activities. EMBO J.

[17] Morishige M (2008). GEP100 links epidermal growth factor receptor signalling to Arf6 activation to induce breast cancer invasion. Nat Cell Biol.

[18] Sabe H (2009). The EGFR-GEP100-Arf6-AMAP1 signaling pathway specific to breast cancer invasion and metastasis. Traffic.

[19] Hashimoto A (2016). P53- and mevalonate pathway-driven malignancies require Arf6 for metastasis and drug resistance. J Cell Biol.

[20] Hashimoto S (2016). Lysophosphatidic acid activates Arf6 to promote the mesenchymal malignancy of renal cancer. Nat Commun.

[21] Menju T (2011). Engagement of overexpressed Her2 with GEP100 induces autonomous invasive activities and provides a biomarker for metastases of lung adenocarcinoma. PLoS One.

[22] Sato H (2014). High level expression of AMAP1 protein correlates with poor prognosis and survival after surgery of head and neck squamous cell carcinoma patients. Cell Commun Signal.

[23] Hashimoto S (2019). ARF6 and AMAP1 are major targets of KRAS and TP53 mutations to promote invasion, PD-L1 dynamics, and immune evasion of pancreatic cancer. Proc Natl Acad Sci U S A.

[24] Makohon-Moore A, Iacobuzio-Donahue CA (2016). Pancreatic cancer biology and genetics from an evolutionary perspective. Nat Rev Cancer.

[25] Aguirre AJ (2003). Activated Kras and Ink4a/Arf deficiency cooperate to produce metastatic pancreatic ductal adenocarcinoma. Genes Dev.

[26] Guerra C (2003). Tumor induction by an endogenous K-ras oncogene is highly dependent on cellular context. Cancer Cell.

[27] Hingorani SR (2003). Preinvasive and invasive ductal pancreatic cancer and its early detection in the mouse. Cancer Cell.

[28] Weissmueller S (2014). Mutant p53 drives pancreatic cancer metastasis through cell-autonomous PDGF receptor beta signaling. Cell.

[29] Onodera Y (2012). Rab5c promotes AMAP1-PRKD2 complex formation to enhance β1 integrin recycling in EGF-induced cancer invasion. J Cell Biol.

[30] Hashimoto A (2016). ZEB1 induces EPB41L5 in the cancer mesenchymal program that drives ARF6-based invasion, metastasis and drug resistance. Oncogenesis.

[31] Onodera Y (2018). Arf6-driven cell invasion is intrinsically linked to TRAK1-mediated mitochondrial anterograde trafficking to avoid oxidative catastrophe. Nat Commun.

[32] Hingorani SR (2005). Trp53R172H and KrasG12D cooperate to promote chromosomal instability and widely metastatic pancreatic ductal adenocarcinoma in mice. Cancer Cell.

[33] Mazaki Y, Hashimoto S, Sabe H (1997). Monocyte cells and cancer cells express novel paxillin isoforms with different binding properties to focal adhesion proteins. J Biol Chem.

[34] Nomi T (2007). Clinical significance and therapeutic potential of the programmed death-1 ligand/programmed death-1 pathway in human pancreatic cancer. Clin Cancer Res.

[35] Yao H (2019). Inhibiting PD-L1 palmitoylation enhances T-cell immune responses against tumours. Nat Biomed Eng.

[36] Thyagarajan A (2019). Myeloid-Deived Suppressor Cells and Pancreatic Cancer: Implications in Novel Therapeutic Approaches. Cancers (basel).

